# The difference in serum proteomes in schizophrenia and bipolar disorder

**DOI:** 10.1186/s12864-019-5848-1

**Published:** 2019-07-11

**Authors:** Liudmila Smirnova, Alexander Seregin, Irina Boksha, Elena Dmitrieva, German Simutkin, Elena Kornetova, Olga Savushkina, Anastasia Letova, Nikolay Bokhan, Svetlana Ivanova, Victor Zgoda

**Affiliations:** 1 Mental Health Research Institute, Tomsk National Research Medical Center of the Russian Academy of Sciences, Tomsk, Russia; 2 Institute of Biomedical Chemistry, Moscow, Russia; 3 Mental Health Research Centre, Moscow, Russia; 4 Siberian State Medical University, Tomsk, Russia

**Keywords:** Biomarker, Bipolar disorder, Schizophrenia, Proteome, Mass spectrometry, Serum

## Abstract

**Background:**

Purpose of study is revealing significant differences in serum proteomes in schizophrenia and bipolar disorder (BD).

**Results:**

Quantitative mass-spectrometry based proteomic analysis was used to quantify proteins in the blood serum samples after the depletion of six major blood proteins. Comparison of proteome profiles of different groups revealed 27 proteins being specific for schizophrenia, and 18 – for BD. Protein set in schizophrenia was mostly associated with immune response, cell communication, cell growth and maintenance, protein metabolism and regulation of nucleic acid metabolism. Protein set in BD was mostly associated with immune response, regulating transport processes across cell membrane and cell communication, development of neurons and oligodendrocytes and cell growth. Concentrations of ankyrin repeat domain-containing protein 12 (ANKRD12) and cadherin 5 in serum samples were determined by ELISA. Significant difference between three groups was revealed in ANKRD12 concentration (*p* = 0.02), with maximum elevation of ANKRD12 concentration (median level) in schizophrenia followed by BD. Cadherin 5 concentration differed significantly (*p* = 0.035) between schizophrenic patients with prevailing positive symptoms (4.78 [2.71, 7.12] ng/ml) and those with prevailing negative symptoms (1.86 [0.001, 4.11] ng/ml).

**Conclusions:**

Our results are presumably useful for discovering the new pathways involved in endogenous psychotic disorders.

**Electronic supplementary material:**

The online version of this article (10.1186/s12864-019-5848-1) contains supplementary material, which is available to authorized users.

## Background

Schizophrenia and BD are the most important mental disorders for social life. They represent a heterogeneous group of endogenous mental disorders with unclarified etiology and pathophysiological mechanisms at present, and these are the causes of difficulties in prediction of responses to treatment and outcomes for the patients with these disorders. Since diagnostics of mental disorders is based only on clinical symptoms, there is a necessity in development of additional methods of biochemical/paraclinical diagnostics. The search for blood based biomarkers which may be used for diagnostics and prognosis of therapy efficacy are very important presently. There are published works devoted to the search for biomarkers of mental disorders in blood serum [1–6]. For instance, 47 proteins are mentioned in a review [6] as possible biomarkers for schizophrenia. The data is described by Sabherwal with co-workers that point to a convergence of pathophysiological mechanisms in schizophrenia that involve the acute-phase response, glucocorticoid receptor signaling, coagulation, and lipid and glucose metabolism [6]. Saia-Cereda with co-workers [7] describe a large amount of proteins typical for brain proteome in schizophrenia, as well as for major depressive disorder (MDD). When compared proteome profiles between schizophrenia, BD, and MDD, only 30 proteins were similarly changed in all three disorders. Hence, small overlapping between changes in protein levels typical for major mental disorders can be a feature maintaining specificity of every disease at proteome level [7].

But most often, when studying the proteomic profile of patients with mental disorders, non-specific proteins are detected. The data confirming dysfunction of intercellular (cell-cell) interactions and blood coagulation in schizophrenia were provided earlier [8, 9]. Influence of treatment with warfarin on remission of psychotic symptoms in patients with schizophrenia is described in a recent study [10]. Other proteome studies in mental disorders also confirm disturbances in these systems [11–13]. Papers devoted to proteome analysis of patients with BD are rare and represented mostly by studies on postmortem brain samples [14, 15]. Some studies of sera from patients with BD have revealed proteins associated with mitochondrial dysfunction and energy metabolism impairment [16–19].

Several studies are known on serum proteomes in BD [20–26] reviewed recently by Preece with co-authors [27], who also have raised the most important challenges in blood proteome biomedical applications, and particularly indicated and explained the causes of disagreements between the results of known studies on BD, such as varying/different proteomic technique, protein quantification method, and the statistical method applied. Besides, patients with BD are characterized by substantial fluctuation of their mental state, and concentrations of disease-associated and other blood proteins can fluctuate as well, so that found “biomarkers” can prove to be state-specific [22] rather than disease-specific.

The proteomics approach enables to specify distinct minor proteins which can help to decipher molecular mechanisms/pathways involved in endogenous mental disorders. However, no protein markers typical only for distinct disease are found still. All the found proteins (and pathways they are involved in) are not specific for pathogenesis of mental disorders. Hence, one may suppose that finally the efforts must not be concentrated on the search for a specific protein, but rather a protein set (panel) must be revealed reflecting the main pathogenetic mechanisms and serving as a starting point for diagnosis and prognosis of mental disorder development.

Serum proteomes of patients with schizophrenia, BD, and healthy subjects are compared in the present study. Besides, the data of quantifications are given for three revealed proteins participating in the most frequently confirmed pathogenetic pathways involved in these disorders, such as cell contacts and endothelial permeability, blood coagulation system, transcription, cell cycle regulation, cell growth and differentiation.

## Methods

### Patients

The following groups of patients were included in the present study: 33 patients (11 men, 22 women) with acute paranoid schizophrenia (F20.0) hospitalized in the Department of Endogenous Disorders of the Mental Health Research Institute (MHRI) at the Tomsk National Research Medical Centre (NRMC, Tomsk), and 23 patients (14 men, 9 women) with bipolar affective disorder (F31), 12 of them were in-patients of the Department of Affective States of MHRI and 11 were in-patients of the Department of Endogenous Mental Disorders and Affective States of the Mental Health Research Centre (MHRC, Moscow). The patients were hospitalized in acute state. Blood was sampled for the study after hospitalization before the beginning of the treatment course. According to anamnestic data, patients did not receive therapy for at least 6 months before hospitalization. The patients were diagnosed by psychiatrists according to the ICD-10. The age of the patients with schizophrenia varied from 22 to 56 years with median of 34 [28, 40] years, and the illness duration was from 2 to 35 years with median of 7 [4, 16] years. The age of patients with bipolar disorder varied from 17 and 62 years, with median of 32 [21, 52] years, and the illness duration was from 4 to 20 years with median of 8 [5, 11] years.

The control group consisted of 24 mentally healthy volunteers without somatic illnesses and other recorded disorders (6 men and 18 women), the group was matched with the studied patient groups by age (median of 28 [21, 55] years) and number of representatives of each gender. The healthy volunteers were selected using special “Questionnaire for the study of healthy persons” developed in MHRI. Some demography data for the studied groups are given in the Table 1 (Results section).

**Table 1:** Demography data for representatives of the studied groups

	Controls	Schizophrenia	Bipolar disorder
Subjects (n)	24	33	23
Age (years)	28 [21;55]	34 [28;40]	32 [21;52]
Gender (M/F)	6/18	11/22	14/9
Duration of illness (years)	–	7 [4;16]	8 [5;11]

Exclusion criteria for all the studied groups were the presence of acute or chronical infections, inflammatory, autoimmune diseases, as well as acute infections no less than 4 weeks before the investigation. The study was carried out according with the Protocol approved by Biomedicine Ethic Committee of TNMRC RAS (Tomsk, Russia) and MHRC (Moscow, Russia) and in accordance with Helsinki Declaration for human experimentation. All the patients and healthy volunteers have signed the Informed Consent for their participation in the clinical trials.

### Sample preparation

Fasting venous blood was collected in the morning in sample tubes (Becton Dickinson Vacutainer, Nederland) containing clot formation activator. Serum was isolated from blood by centrifugation for 20 min at 2000×*g* using the Digicen 21R centrifuge (Orto Alresa, Spain). Then serum was aliquoted and stored at − 80 °C.

Six major high-abundance proteins – serum albumin, IgG, IgА, antitrypsin, transferrin, and haptoglobin were depleted from the sera before the further studies. For this, the samples were five-fold diluted with PBS, centrifuged (4000 g, 4 °C, 5 min) using Zentrifuge Z 36 HK centrifuge (Hermle labortechnik Gmbh, Germany), filtered through Filtropur S 0.2 membrane (Sarstedt), and the supernatant was then passed through the Multiple Affinity Removal Column Human 6 (4.6 × 100 mm, Agilent, USA) for affinity binding of the major proteins, with subsequent concentration of the column flow-through by ultrafiltration through 5 kDa Microcon® Centrifugal Ultrafilters (Millipore, France) in accordance with the protocol provided by the manufacturer. Protein concentration was measured by absorbance at 280/260 nm using Epoch microplate spectrophotometer (BioTek, USA) with the software installed.

### One-dimensional Laemmli PAG electrophoresis

Reducing Laemmli sample buffer (1,1 v/v) was added to all the samples pre-treated as described above, and the samples were heated at 95 °C for 5 min and centrifuged briefly to precipitate the condensate. The samples matched by the total protein content of 20 μg (respective volumes of samples were calculated for this) were subjected to the sodium dodecyl sulfate polyacrylamide gel electrophoresis by Laemmli in 12% PAG (1 mm gel thickness, 160 mm x 160 mm dimensions) [28]. The electrophoretic separation procedure was carried out using Protean II xi Cell (Bio-Rad, USA) device at 150–180 V supplied by the PowerPac™ Universal Power Supply source (Bio-Rad, USA).

The gels were stained with Coomassie Brilliant Blue G250 (0,1% Coomassi Brilliant Blue G250, 40% C_2_H_5_OH, 10% CH_3_COOH), then destained in solution with 70% C_2_H_5_OH for 2 h.

Molecular masses corresponding to the stained protein bands in PAGs were automatically calculated using Alliance 2.7 Uvitec system (Cambridge) with the supplied software, relatively to molecular masses of protein standards (Broad range, Fermentas, Thermo Fisher Scientific). Significant differences were identified using the Fisher’s exact test with Yates’ correction. Protein bands significantly more frequently met in gel lanes corresponding to patients with schizophrenia or bipolar disorder in comparison with lanes corresponding to control subjects (where they were virtually absent) were subjected to further analysis. The 5–7 protein bands were usually analyzed for each person. Thus, not all bands were included in the study.

### Protein identification and quantitation

Protein bands were cut from PAG manually (volume of ~3mm^3^) using scarifier, placed into microtubes, and incubated with 50 mM NH_4_HCO_3_ in 50% acetonitrile for 10–15 min on a shaker to remove Coomassie staining. The supernatant was discarded, and the PAG was washed with deionized water. The procedure was triplicated or more (till complete destaining). Then the samples were liophilized for 45 min at 40°С. In-gel trypsinolysis of the proteins was carried out using Sequencing Grade Modified Trypsin (#V511A, Promega, USA) diluted by the supplied solution (50 mM CH_3_COOH) and then, sequentially, by 50 mM NH_4_HCO_3_ pH = 8, to the concentration of 0.01–0.025 μg/ml. Twenty microliters of the trypsin solution were added to every sample and incubated at 4 °C for 1 h for the gel swelling. The samples where then incubated at 37 °C for 18 h for trypsinolysis. After finishing the reaction, 25 mM NH_4_HCO_3_ was added to every sample and shaken on Vortex, and supernatants were placed in separate tubes. Then further extraction of peptide mixtures from the gels was done with 50% acetonitrile in 5% formic acid, the procedure was triplicated. The extracts were lyophilized and frozen. To estimate the amount of protein there were used exponentially modified PAI (emPAI), defined as the number of identified peptides, divided by the number of theoretically observed tryptic peptides for each protein [29, 30].

Peptide analysis by mass-spectrometry was carried out in IBMC, Moscow (Centre of Collective Usage “Human proteome”). The peptide samples obtained were analyzed using the Agilent HPLC system1100 Series (Agilent Technologies) connected to a hybrid linear ion trap LTQ Orbitrap Velos (Thermo Fisher Scientific), equipped with a nanoelectrospray ion source (Thermo Scientific). Peptide separations were carried out on an RP-HPLC Zorbax 300SB-C18 column (C18 3.5 μm, 75 μm inner diameter and 150 mm length) using a linear gradient from 95% solvent A (water, 0.1% formic acid) and 5% solvent B (water, 0.1% formic acid, and 80% acetonitrile) to 60% solvent B over 45 min at a flow rate of 0.3 μl/min.

Mass spectra were acquired in the positive ion mode using Orbitrap analyzer with a resolution of 30,000 (m/z 400) for MS and 7500 (m/z 400) for MS/MS scans. The AGC target was set at 2 × 10^5^ and 1 × 10^5^ with maximum ion injection time 50 and 100 ms for MS and MS/MS level, respectively. Survey MS scan was followed by MS/MS spectra of the five most abundant precursors. The higher energy collisional dissociation (HCD) was used, the signal threshold was set to 5000 for an isolation window of 2 m/z. The normalized collision energy was set to 35 eV. The precursors fragmented were dynamically excluded from targeting with repeat count 1, repeat duration 10 s, and exclusion duration 60 s. Single charged ions and those with not defined charge state were excluded from triggering the MS/MS scans [31].

The mass spectrometric raw data were analyzed with the MaxQuant software (version 1.6.3.4). Default parameters were used unless otherwise specified below. A false discovery rate (FDR) of 0.01 for proteins and peptides and a minimum peptide length of 6 amino acids were required. The mass accuracy of the precursor ions was improved by the time-dependent recalibration algorithm of MaxQuant. Match between runs options was set to allow transferring identification across the runs. The Andromeda search engine was used to search the MS/MS spectra against the Uniprot human database (containing 90,482 entries, download date 2019 − 01-17) combined with 262 common contaminants and concatenated with the reversed versions of all sequences. Enzyme specificity was set to trypsin specificity, allowing cleavage N-terminal to proline. Further modifications were cysteine carbamidomethylation (fixed) as well as protein N-terminal acetylation, asparagine and glutamine deamidation and methionine oxidation (variable). A maximum of two missed cleavages were allowed. Peptide identification was based on a search with an initial mass deviation of the precursor ion of up to 7 ppm. The fragment mass tolerance was set to 20 ppm on the m/z scale. Only proteins quantified with at least two peptides were considered for quantitation.

MS1-intensity based label-free quantitation was used to assess differences in the abundance of proteins between all the studied groups. LFQ intensities for the proteins were log2-transformed and normalized to ensure equal median protein abundance across the samples. A two-tailed unpaired t-test with an FDR value of 0.05 and S0 = 2 was applied to identify proteins for which the abundance was significantly changed between all the studied groups [32].

### ELISA

All the target proteins were detected by commercially available ELISA kits representing sandwich enzyme immunoassays for in vitro quantitative measurements of cadherin 5 and ANKRD12 in human serum according with the manufacturer protocols.

### ELISA of cadherin 5

Sample preparation: for quantitative measurement of cadherin 5 concentration every serum sample was diluted 1.5 folds (100 μl serum + 50 μl PBS). Concentration of cadherin 5 was determined using SEB366Hu 96 Tests ELISA Kit for cadherin 5 (CDH5) from *Homo sapiens* (Human) (Cloud-Clone Corp., USA) with the detection range of 78–5000 pg/ml and minimum detectable concentration (sensitivity) of 29 pg/ml.

### ELISA of Ankyrin repeat domain protein 12

Sample preparation: A preliminary experiment showed that the ANKRD12 concentration was within the range of the kit determination, and the serum samples were not diluted but used directly for analysis. Concentration of ANKRD12 was determined using SEM789Hu 96 Tests ELISA Kit for ANKRD12 from *Homo sapiens* (Human) (Cloud-Clone Corp., USA) with the detection range of 0.312–20 ng/ml and the sensitivity of 0.112 ng/ml.

### Statistical analysis

Statistical analysis was performed using the Statistica version 10.0 (StatSoft, Tulsa, OK, USA). The Kolmogorov–Smirnov test was used to determine whether the data were normally distributed. Non-parametric Mann Whitney U-test and Fisher’s exact test with Yates’ correction was used to check the statistical significance of between-group differences. Kruskal-Wallis оne-way analysis of variance (ANOVA) and Median test were used in comparison data for three groups. Spearman rank test was used to search for correlations. Differences and correlations were considered significant at *p*-value   <   0.05.

## Results and discussion

### Differences in serum protein sets between schizophrenia and BD

All the subjects included in the study comprised three groups, namely patients with schizophrenia, BD, and healthy controls, matched by age, gender, and (for patients) by illness duration (Table 1). When the groups were compared pairwise, no significant between-group differences were found in age: *p* = 0.35 for patients with schizophrenia and controls, *p* = 0.83 for patients with BD and controls, and *p* = 0.18 for patients with BD and schizophrenia.

No significant difference was found between patients with schizophrenia and BD in the illness duration (*p* = 0.69). Non-parametric statistics was used here and everywhere further, because not always the data were distributed normally.

Ten representatives of every group were randomly selected from each the group for mass-spectrometry analysis. In total, about 1600 proteins were identified for each person. Bioinformatics approach using MaxQuant version 1.6.3.4. applied to the group of patients with schizophrenia and to the group of patients with BD has resulted in identification of unique proteins that have not been met in other groups. The resulting t-test -significant proteins for each group are presented in Tables 2 and 3. In addition, we compared proteins in patients with schizophrenia and BD with a database of plasma proteins from the Human Plasma Proteome Project (http://www.peptideatlas.org/hupo/hppp/). Proteins found in patients are not represented in these databases.

**Table 2:** Proteins found in serum from patients with schizophrenia and absent in samples from controls and patients with BD

Protein IDs	Protein names	Gene names	iBAQ	LFQ intensity
A8K2U0	Alpha-2-macroglobulin-like protein 1	A2ML1	381,783	5,580,283
O75820	Zinc finger protein 189	ZNF189	4,301,833	5,612,922
O95347	Structural maintenance of chromosomes protein 2	SMC2	4,376,279	4,833,428
P00748	Coagulation factor XII	F12	4,003,762	5,272,229
P01011	Alpha-1-antichymotrypsin	SERPINA3	6,461,004	799,853
P02649	Apolipoprotein E	APOE	4,510,154	6,384,497
P02750	Leucine-rich alpha-2-glycoprotein	LRG1	4,698,267	6,302,049
P05154	Plasma serine protease inhibitor	SERPINA5	5,085,402	6,709,058
P11532	Dystrophin	DMD	3,935,035	5,537,725
P22792	Carboxypeptidase N subunit 2	CPN2	46,868	6,312,685
P42684	Tyrosine-protein kinase ABL2 (Abelson tyrosine-protein kinase 2)	ABL2	5,750,121	8,166,816
P60709	Actin, cytoplasmic 1	ACTB	4,478,685	594,762
P63261	Actin, cytoplasmic 2	ACTG1	4,478,685	594,762
P78527	DNA-dependent protein kinase catalytic subunit	PRKDC	5,092,892	4,734,974
P81605	Dermcidin	DCD	5,598,764	6,677,141
P84098	Ribosomal protein L19	RPL19	5,175,687	6,112,823
P98164	Low-density lipoprotein receptor-related protein 2	LRP2	4,171,199	5,510,686
Q08380	Galectin-3-binding protein	LGALS3BP	43,327,882	5,893,399
Q15811	Intersectin-1	ITSN1	4,663,725	461,726
Q16610	Extracellular matrix protein 1	ECM1	5,750,121	8,166,816
Q5H9R4	Armadillo repeat-containing X-linked protein 4	ARMCX4	370,518	5,322,898
Q6UB98	Ankyrin repeat domain-containing protein 12	ANKRD12	4,403,913	594,308
Q7Z478	ATP-dependent RNA helicase DHX29	DHX29	4,348,332	458,995
Q8TE73	Dynein heavy chain 5, axonemal	DNAH5	3,785,117	6,673,067
Q96BK5	PIN2/TERF1-interacting telomerase inhibitor 1	PINX1	5,743,424	6,480,803
Q96KN2	Beta-Ala-His dipeptidase	CNDP1	4,732,742	6,322,812
Q9UGM5	Fetuin-B	FETUB	5,034,075	5,770,932

**Table 3:** Proteins found in serum from patients with BD and absent in samples from controls and patients with schizophrenia

Protein IDs	Protein names	Gene names	iBAQ	LFQ intensity
O15417	Trinucleotide repeat-containing gene 18 protein	TNRC18	3,960,068	4,697,438
O95445	Apolipoprotein M	APOM	5,199,102	6,902,737
P02666	Beta-casein	CSN2	461,475	6,030,776
P02745	Complement C1q subcomponent subunit A	C1QA	5,177,613	5,991,639
P02753	Retinol-binding protein 4	RBP4	4,882,314	6,522,907
P05090	Apolipoprotein D	APOD	4,731,677	63,181
P05452	Tetranectin	CLEC3B	4,780,069	6,317,698
P07360	Complement component C8 gamma chain	C8G	459,154	5,882,624
P13671	Complement component C6	C6	4,384,574	6,239,425
P15924	Desmoplakin	DSP	3,736,801	5,864,175
P17948	Vascular endothelial growth factor receptor 1	FLT1	4,665,292	6,331,822
P23141	Liver carboxylesterase 1	CES1	3,948,734	6,577,147
P33151	Cadherin-5	CADH5	4,472,995	6,134,045
P46013	Antigen KI-67	MKI67	4,093,921	5,578,263
Q01538	Myelin transcription factor 1	MYT1	607,717	8,153,738
Q86YZ3	Hornerin	HRNR	5,692,768	7,672,241
Q9HCI5	Melanoma-associated antigen E1	MAGEE1	4,417,353	5,745,221
Q9UBP9	PTB domain-containing engulfment adapter protein 1	GULP1	5,111,498	6,666,724

### Biological functions of found proteins

Proteins uniquely met only in sera from patients with schizophrenia are presented in the Table 2. These proteins are involved mostly in biological processes, such as protein metabolism and cell communication, followed by immune response, regulation of nucleic acid metabolism, cell growth and maintenance. By their molecular weights, most of the proteins were within the ranges designated in previously published work [33] showing significant differences when patterns in 1-D PAGE of serum proteins of patients with schizophrenia and healthy subjects were compared. The revealed proteins met in sera of patients with mental disorders were classified in accordance with the biological/molecular processes/pathways using Human Protein Reference Database [http://www.hprd.org/].

The proteins identified in this work can directly participate in various pathogenetic processes in schizophrenia. Extracellular matrix protein 1, and Abelson tyrosine protein kinase 2 participate in cell communication and signaling, particularly, in actin-dependent signaling, which has recently received much attention in the development of diseases with an inflammatory component [34, 35]. Besides, Abelson tyrosine-protein kinase 2 can fulfill an important role because it can regulate neurotransmission in the brain via protein phosphorylation in synapses [36]. We have also discovered actin, cytoplasmic 1 and actin, cytoplasmic 2 that participate in many biological functions. Extracellular actin can be involved in the development of pathologies as an inducer of autoimmunity, induction of death of endotheliocytes, reduced ability of the actin clearance system to sequester inflammatory mediators [37–39]. An interesting fact is that actin in complex with the cell surface is the center of plasminogen binding, involving actin in the processes of angiogenesis and modulation of neurotransmission [40–42]. Zinc finger protein is known to be a transcription factor playing an important role in brain development, as well as in development of mental and cognitive disorders [43, 44].

Proteins uniquely met only in sera from patients with BD are presented in the Table 3. The unique proteins identified in sera from patients with BD mainly participate in regulation of DNA synthesis and cell cycle, particularly in differentiation of neural progenitor cells, development of neurons and oligodendrocytes, such as myelin transcription factor regulating genes encoding myelin associated proteins and other proteins, followed by immune response, regulating transport processes across cell membrane and cell communication [45–49].

**Fig. 1:**
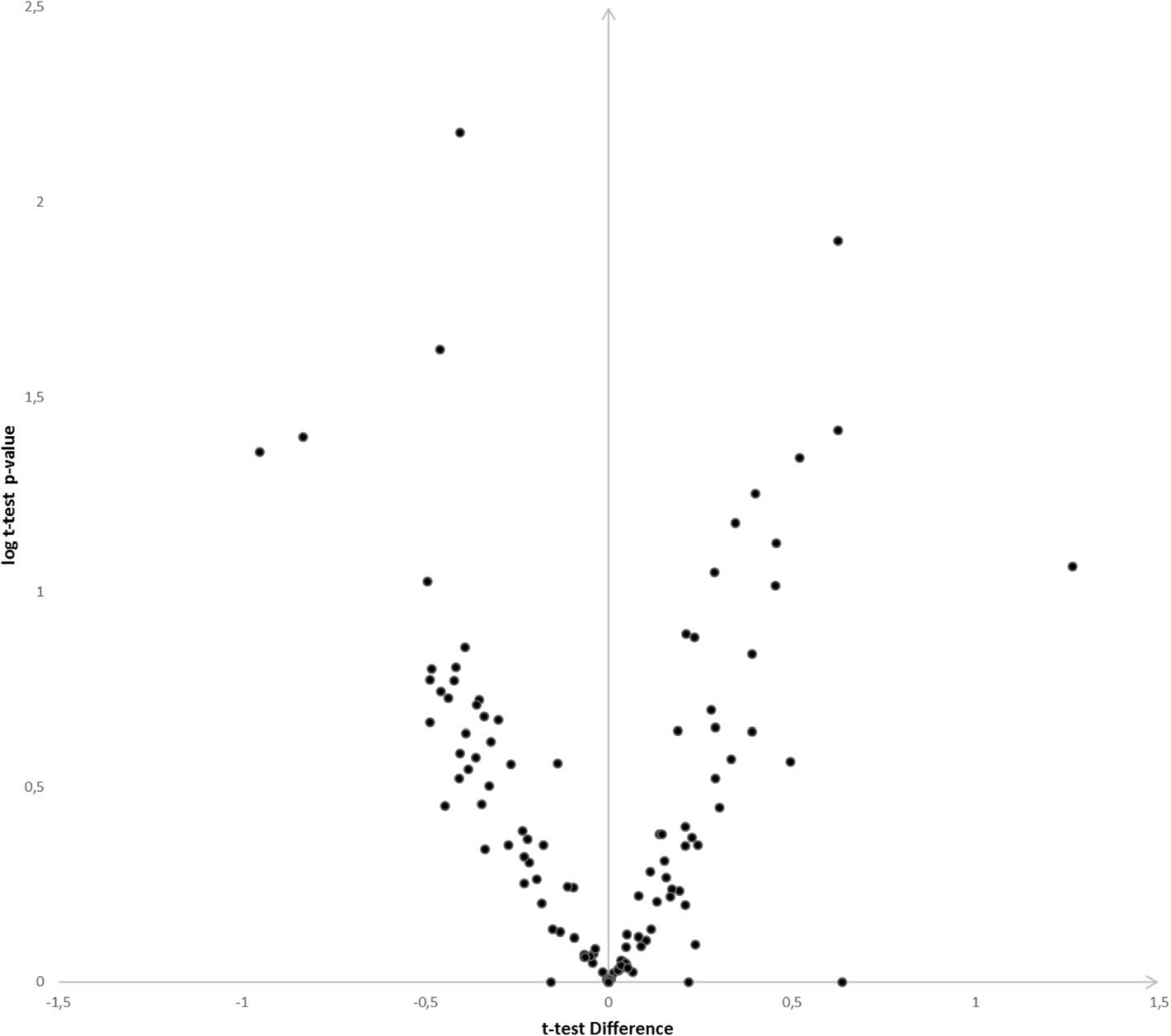
Label-free proteome quantification of bipolar disorder and schizophrenia. The ordinate indicates-lg(p), where *p* is *p*-value in *t*-test. The abscissa axis shows the ratio of proteins to bipolar disorder against schizophrenia (mean (iBAQ bipolar disorder) – mean (iBAQ schizophrenia))

Figure 1 shows representative volcano plot of protein abundance differences as a function of statistical significance (t-test *p* ≤ 0.05 and fold change cutoff point ±2) between bipolar disorder and schizophrenia.

Ankyrin repeat domain-containing proteins are one of the most common amino acid sequence motifs that mediate interactions between proteins and nearly every cellular process from transcriptional regulation in the nucleus to cell adhesion at the plasma membrane [50]. This motifs are associated with a number of human diseases including cancer (p16 protein) [51] neurological disorders (Notch protein) [52], and skeletal dysplasias (TRPV4 protein) [53], and variations in the amino acid sequence of the human ANKK1 are associated with addictive behaviors such as alcoholism and nicotine addiction [54, 55]. The functions of the Ankyrin repeat domain-containing protein 12 (ANKRD12 or ANCO-2) remain unclear, however, it is assumed that it plays the role in recruiting HDACs to the p160 coactivators/nuclear receptor complex to inhibit ligand dependent transactivation [56]. HDACs are playing an important role in regulation of gene expression. Elevated HDAC1 expression in prefrontal cortex and hippocampus was revealed in postmortem studies of brain from patients with schizophrenia [57, 58]. Also HDAC1 and HDAC2 expression was linked to mechanisms of schizophrenia, BD, and depression [59–61]. Elevation of HDAC activity in parallel with enhancement of depressive symptoms and decreased response to antidepressants were discovered using animal models [62, 63]. Proteins with ankyrin repeat domains participate in regulation of various protein-protein interactions and fulfill structural functions in CNS in the subcellular structures of neurons, such as the axon initial segment and nodes of Ranvier, in which ankyrins regulate the localization of ion channels. Association of ankyrin 3 (ANK3) gene variants (ANK3 is a large gene encoding multiple isoforms of the ankyrin G protein) with BD suggests a link between ankyrin repeat domain proteins and BD, however pathogenetic mechanisms of this link are not clear yet [64]. Besides, mutations in ANK3 described by Lopez can cause hypersensitivity of neurons to excitating stimuli [65]. The described above data was the reason for more detailed study of quantitative changes in Ankyrin repeat domain-containing protein 12 (ANKRD12) by ELISA in all groups included in the study.

Also the subject of our further study was cadherin 5. Cadherin protein family performs not only mechanical contact between neighboring cells, but also participates in intracellular signaling regulating processes of proliferation, migration, cell sorting, differentiation, and morphogenesis [66, 67]. In tissues of adult organisms cadherins regulate the renewal of cell composition, provide a physiological barrier between contacting tissues and selectivity of transport of soluble substances. Some inflammatory response mediators, such as thrombin, bradykinin, histamine, vascular endothelial growth factor, etc. when binding their receptors can disrupt the organization of contacts, thereby opening the barrier, and plasma proteins can pass through the endothelial barrier [68, 69]. Particularly, the protein vascular endothelial growth factor receptor 1 (VEGFR1) found in BD participates in the initiation of autophosphorylation of cadherin 5 signal cascades directly influencing the development of endothelial dysfunction [70]. Besides cadherins are known to mediate cell sorting, migration and segregation, morphogenesis and axonal growth in embryogenesis [71, 72]. Wang and co-authors have demonstrated involvement of genes encoding CDH9 and CDH10 family in autism disorder [73]. These genes participate in neural cell adhesion [74]. This fact enables to suppose existence of defects in neural cell adhesion in autism [75]. However, no data are known yet suggesting participation of cadherin 5 in pathogenesis of mental disorders.

### ELISA of ANKRD12 and cadherin 5

The levels (concentrations) of these candidate proteins (Ankyrin repeat domain-containing protein 12 and cadherin 5) were measured in sera from the studied groups (controls, Ctr, bipolar disorder, BD, and schizophrenia, SCH) by ELISA using commercially available kits. Of two studied proteins significant between-group differences were found only for ANKRD12 concentrations by Kruskal-Wallis test (*p* = 0.02) and by Median test (χ2 = 6.97, *p* = 0.03) (Table 4).

**Table 4:** The levels Ankyrin repeat domain-containing protein 12 and Cadherin-5 in sera from the studied groups

	Schizophrenia		Bipolar disorder		Control		Kruskal-Wallis	Median
	Me [Q_25_;Q_75_]	N	Me [Q_25_;Q_75_]	N	Me [Q_25_;Q_75_]	N	test, p	Test, p
Ankyrin repeat domain-containing protein 12, ng/ml	0.92 [0.01;1.52]	33	0.01 [0.01;0.57]	23	0.01 [0.01;0.70]	24	0.02	0.03
Cadherin 5, ng/ml	2.70 [0.40;5.40]	33	3.51 [2.09;6.35]	23	2.73 [1.91;4.84]	24	0.42	0.55

**Fig. 2:**
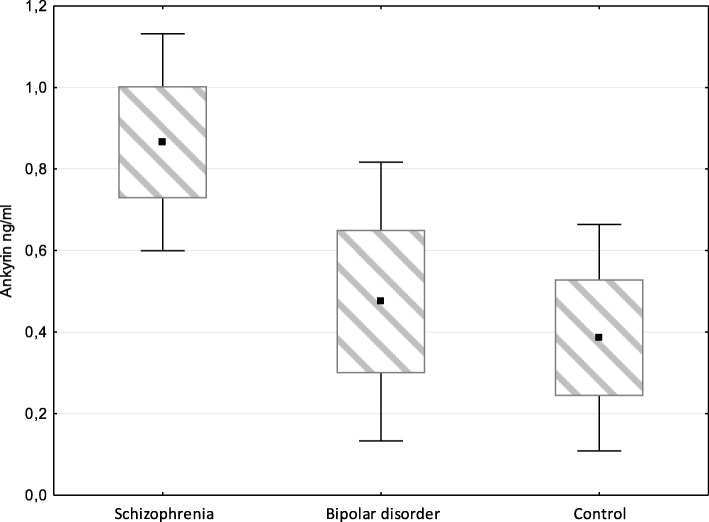
ANKRD12 concentrations measured in groups of patients with schizophrenia or bipolar disorder, and in controls

When measuring ANKRD12 concentrations the lowest median was found in Ctr group, intermediate – in BD group, and maximum in SCH group. So, median value for ANKRD12 concentrations comprised 0.38 ng/ml, with measured (i.e. within the detectable range) concentrations ranging from 0.44 ng/ml to 2.2 ng/ml in sera of Ctr; the respective values comprised 0.47 ng/ml (from 0.27 ng/ml to 2.96 ng/ml) in BD group, and 0.87 ng/ml (from 0.37 to 2.43 ng/ml) in SCH group (Fig. 2).

Then pairwise comparison of the groups was done by Mann-Whitney U-test, and difference was assigned significant at *p* < 0.05. For SCH-Ctr pair the Z-Score = 2.4, the *p*-value was 0.02, and the result was significant. For SCH-BD pair the Z-Score = 2.2, the p-value was 0.045, the result was significant. For BD-Ctr pair the Z-Score = 0.17, the p-value was 0.67, the result was not significant.

Ankyrin repeats are the most widespread structural domains amongst eukaryotic proteins. Proteins with ankyrin repeats are found to be intracellular and extracellular [76, 77]. Each the repeat consists of two antiparallel α-helices and a prolonged loop terminating with β-hairpin [78]. Multiple repeats form a specific structure in which a core is formed by inter-helix interactions, and termini of β-hairpins are displayed outwards and represent potential sites for protein-protein interactions. Ankyrin repeats indirectly participate via the protein-protein interactions in various cellular functions, such as transcription, cell cycle regulation, cell differentiation, apoptosis, ion transfer, signal transduction, etc. [79], and in initiation/development of immune response in eukaryotes [53], and these functions are provided by definite protein spatial conformation, but not by amount of the repeats [80].

ANKRD12 and its related protein ANCO-1 are nuclear proteins which may recruit histone deacetylases (HDACs) to the p160 coactivators/nuclear receptor complex to inhibit ligand-dependent transactivation. ANCO-2 may represent a novel class of nuclear receptor corepressors that may inhibit transcriptional activity of NRs through interfering with the coactivator function of p160 by recruiting HDACs. Regulation of histone acetylation by HDAC inhibitors was found to be enhancing memory formation processes [81], namely HDAC2 was shown to play as a negative regulator for memory and synaptic plasticity, thus influencing cognitive functioning [82, 83]. Enhancement of HDAC activity may cause manifestation of various clinical symptoms such as cognitive impairment and negative symptoms [61].

Many studies suggest a link between ANCO-1 and impaired synaptic function and development of neurons. For instance, carriers of mutant ANCO-1 have cognitive disorders and neuroanatomical anomalies [84]. Being a transcriptional co-regulator, ANCO-1 influences multiple genes linked to autistic spectrum disorders, many of them are related to SCH [84–86]. Basing on the mechanisms proposed by Zhang et al. [56], one can admit that ANCO-2 plays a role in SCH development as well, especially taking into account significant between-group differences in ANCO-2 levels found in the present work. In addition, one may admit that depressive episode diagnosed in almost all BD patients included into the study is associated with a tendency to elevation of ANCO-2 level revealed in sera from these patients.

Spearmen rank order correlation analysis did not reveal any link between the levels of the revealed proteins and age or gender in general population of studied subjects (data not given). No significant correlation was found for the investigated parameters between each other and depending on age in the group of healthy persons (Table 5), or in patients with BD (Table 6). However, the ANKRD12 levels were found to be dependent on the patient’s age in the schizophrenic group (Table 7).

**Table 5:** Spearman Rank Order Correlations in the control group

	Valid - N	Spearman - R	p-value
ANKRD12, ng/ml & Age	24	−0,10	0,67
Cadherin 5, ng/ml & Age	24	0,06	0,80
ANKRD12, ng/ml & Cadherin, 5 ng/ml	24	−0,28	0,24

**Table 6:** Spearman Rank Order Correlations in group of patients with BD

	Valid - N	Spearman - R	p-value
ANKRD12, ng/ml & Age	23	0,16	0,45
Cadherin 5, ng/ml & Age	23	-0,22	0,32
АKRD12, ng/ml & Cadherin, 5 ng/ml	23	0,17	0,44

**Table 7:** Spearman Rank Order Correlations in the group of patients with schizophrenia

	Valid - N	Spearman - R	p-value
ANKRD12, ng/ml & Age	33	-0,35	0,04
Cadherin 5, ng/ml & Age	33	0,04	0,83
ANKRD12, ng/ml & Cadherin, 5 ng/ml	33	-0,18	0,97

Thus, a significant negative correlative link between ANKRD12 concentration and age was observed in patients with schizophrenia (R = − 0.35 *p* = 0.04). It is not excluded that the age-related changes in the concentration of protein appear against the background of long-term treatment of this group of patients, throughout their life, with neuroleptics.

**Fig. 3:**
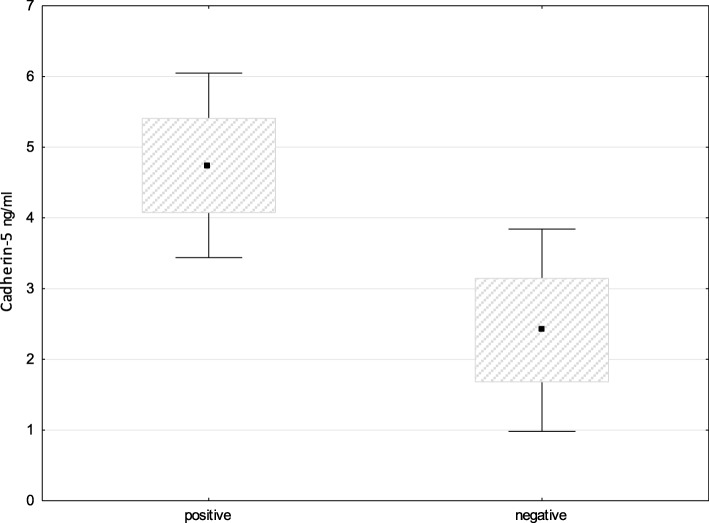
Levels of cadherin 5 in subgroups of patients with schizophrenia with prevailing positive or negative symptoms. Pairwise comparison of the groups by Mann-Whitney U-test reveals a significant difference

As a result of our study, the most numerous family of proteins presented in both groups of patients (with schizophrenia and BD) are the proteins of cell skeleton and adhesion. However, cadherin 5 was not covered in these studies. The first main function of VE-cadherin is to maintain the proper assembly of adhesive contacts at the initial stages of vascular development and to provide the normal functioning of the endothelial barrier [87]. Several works were published recently confirming the blood-brain barrier (BBB) damage in patients with endogenous psychoses [88, 89]. When analyzing the content of cadherin 5 in newborns with perinatal CNS lesion, endothelial dysfunction and BBB damage were revealed. The protein was proposed as a marker of the above listed disorders [34]. The absence of significant differences in the concentration of cadherin 5 among the studied groups was initially the object of our disappointment. However, since any significant difference was not revealed in comparative between-group studies we searched for links with clinical data for the patients. For comparison, we used a known indicator in psychiatric practice such as an estimation of prevailing positive or negative symptoms in patients with schizophrenia. In fact, the significant difference was found in levels of cadherin 5 between subgroups of patients with schizophrenia with prevailing positive or negative symptoms (*p* = 0.035) (Fig. 3). The patients with prevailing negative symptoms are assessed as patients with a heavier course of the disease and a poor prognosis of response to pharmacotherapy. Hence, the determination of the concentration of cadherin 5 in serum of patients with schizophrenia has definite prospects for improving the diagnosis of mental disorders.

### Comparative findings in serum proteome research in schizophrenia and BD

We did some benchmarking against the markers discussed in the recent review of Preece et al., 2018 [25]. Several studies are known on serum proteomes in BD [20–25] reviewed recently by Preece with co-authors [27]. Song with co-authors [21] performed a comparative proteomic study to identify differentially expressed plasma proteins in various BD mood states (depressed, manic, and euthymic) relative to controls. Their technique was similar to ours in depleting high-abundance proteins in plasma samples, but differed from ours in using 2-D instead of 1-D electrophoresis, thus, the obtained results can be scarcely compared, but, nevertheless, three common proteins were identified.

Using serum and plasma in multiplex immunoassay analyses of 190 proteins and small molecules, Alsaif with co-authors [24] tested whether differences between serum and plasma can influence the identification of potential biomarkers in a preliminary study comparing BD patients with controls, and the remark was made, such as “important differences in inter individual variability can have significant impact on identifications made in biomarker studies”. Haenisch with co-authors [25] have investigated the utility of a biomarker panel as a diagnostic test for BD and identified 20 protein disease-specific analytes with predictive performance, although no further studies have been published yet reproducing or supporting these findings.

A number of protein markers listed in review of Preece et al. [27] are also presented in our tables generated as a result of the data processing using bioinformatics methods. We are regarding those proteins which are found in patients inspected in our study. There are published data [90–92] on the increase in Apolipoprotein D levels in patients with major depression (MD) in comparison with controls. In our study the elevated level of Apolipoprotein D is found also in patients with bipolar disorder (BD). The opposite regularity was found in the level of another apolipoprotein. In fact, also in line with our results, [93] has found decreased level of Apolipoprotein E in patients with BD in comparison with controls.

We have revealed the increase in Alpha-1- antichymotrypsin in patients with schizophrenia, but not in BD. There are evidences on the increase in Alpha-1- antichymotrypsin level [91], and Hornerin [92] in patients with MD.

Lee et al. [94] have found the increase in levels of Complement C1q subcomponent subunit C in patients with MD in comparison with controls, besides, Song et al. [22] have found the increase in the level of this protein in patients with BD as compared with controls. In agreement with our data, this protein is also elevated in patients with BD.

The data on the levels of Alpha-2-macroglobulin in mental disorders vary substantially in various publications. The increase in Alpha-2-macroglobulin level in patients with MD was found by [90, 95, 96]. Besides, Alsaif et al. [24] and Song et al. [22] have found the decrease in patients with BD in comparison with healthy controls. This is in agreement with our results.

The increase in levels of Retinol-binding protein 4 [23] and decrease in Carboxypeptidase N [22] have been found in patients in BD compared with controls that is also in agreement with our results.

Chen et al. have found the decrease in Actin, cytoplasmic 2 protein in patients with MD in comparison with BD [2]. We have revealed the increase in levels of actin group proteins in patients with schizophrenia in comparison with BD patients.

## Conclusions

As a result of the mass-spectrometry analysis, from 50 to 350 proteins were identified in every band, and about 1600 proteins were identified for each person. Comparison of proteome profiles of different groups revealed 27 proteins being specific for schizophrenia, and 18 – for BD. Protein set in schizophrenia was mostly associated with immune response, cell communication, cell growth and maintenance, protein metabolism and regulation of nucleic acid metabolism. Protein set in BD was mostly associated with immune response, regulating transport processes across cell membrane and cell communication, development of neurons and oligodendrocytes and cell growth. Many of these pathways are involved in the pathogenesis of mental disorders. They are interesting as potential markers, although some of them are already represented in proteomic studies in other diseases [35, 97–99].

Concentrations of ankyrin repeat domain-containing protein 12 (ANKRD12) and cadherin 5 in serum samples were determined by ELISA. Significant difference between three groups was revealed in ANKRD12 concentration (*p* = 0.02), with maximum elevation of ANKRD12 concentration (median level) in schizophrenia. Ankyrin repeat domain-containing protein 12 (ANKRD12) is a nuclear protein which may recruit histone deacetylases (HDACs) to the p160 coactivators/nuclear receptor complex to inhibit ligand-dependent transactivation. Regulation of histone acetylation by HDAC inhibitors was found to be enhancing memory formation processes [81], namely HDAC2 was shown to play as a negative regulator for memory and synaptic plasticity, thus influencing cognitive functioning [82, 83]. Elevated HDAC1 expression in prefrontal cortex and hippocampus was revealed in postmortem studies of brain from patients with SCH. Enhancement of HDAC activity may cause manifestation of various clinical symptoms such as cognitive impairment and negative symptoms [61]. Thus, as a result of our study, a new potential protein marker was found allowing indirect assessment the state of cognitive functions and synaptic plasticity among patients with schizophrenia. More definite mechanisms influencing the increase of ANKRD12 concentration in serum of patients with schizophrenia required clarification. The decrease of ANKRD12 concentration with the increase of patient’s age was discovered by correlation dependence of moderate force in the same group of patients. For diagnostic purpose interesting and prospective finding was the discovery of Cadherin 5 concentration differed significantly between schizophrenic patients with prevailing positive symptoms (4.78 [2.71, 7.12] ng/ml) and those with prevailing negative symptoms (1.86 [0.001,4.11] ng/ml) (*p* = 0.035). Thus, our results are presumably useful for discovering the new pathways involved in schizophrenia and BD.

## Additional files

Additional file 1: Approval by IRB-Ru. (PDF 3580 kb)

Additional file 2: Approval by IRB.-Eng. (PDF 217 kb)

Additional file 3: A statement regarding patient consent. (PDF 108 kb)

## Data Availability

The mass spectrometry proteomics data have been deposited to the ProteomeXchange Consortium via the PRIDE partner repository with the dataset identifier PXD009219.

## Abbreviations

ANCO-1: Ankyrin Repeat Domain-Containing Protein 11
ANCO-2: Ankyrin Repeat Domain-Containing Protein 12
ANK3: Ankyrin 3
ANKK1: Ankyrin repeat and kinase domain containing 1
ANKRD12: Ankyrin Repeat Domain Protein 12
BBB: Blood-brain barrier
BD: Bipolar disorder
CDH10: Cadherin 10
CDH9: Cadherin 9
CNS: Central nervous system
DNA: Deoxyribonucleic acid
FDR: False discovery rate
HCD: Higher energy collisional dissociation
HDAC: Histone deacetylases
HPLC: High performance liquid chromatography
iBAQ: Intensity Based Absolute Quantification
IBMC: Institute of Biomedical Chemistry
ICD-10: The International Statistical Classification of Diseases and Related Health Problems 10th revision
LFQ: Label-free quantification
MD: Major depression
MDD: Major depressive disorder
MHRC: Mental Health Research Centre
MHRI: Mental Health Research Institute
PAG: Polyacrylamide gel
PBS: Phosphate buffered saline
SCH: Schizophrenia
TNRMC RAS: Tomsk National Research Medical Centre Russian Academy of Sciances
TRPV4: Transient receptor potential cation channel subfamily V member 4
VEGFR1: Vascular endothelial growth factor receptor 1

## References

[1] Ding YH, Guo JH, Hu QY, Jiang W, Wang KZ (2015). Protein Biomarkers in Serum of Patients with Schizophrenia. Cell Biochem Biophys..

[2] Chen J, Huang C, Song Y, Shi H, Wu D, Yang Y, Rao C, Liao L, Wu Y, Tang J, Cheng K, Zhou J, Xie P (2015). Comparative proteomic analysis of plasma from bipolar depression and depressive disorder: identification of proteins associated with immune regulatory. Protein Cell..

[3] Steiner J, Guest PC (2047). A Clinical Study Protocol to Identify Serum Biomarkers Predictive of Response to Antipsychotics in Schizophrenia Patients. Adv Exp Med Biol..

[4] Ivanova SA, Boyko AS, Fedorenko OY, Krotenko NM, Semke AV, Bokhan NA (2014). Glutamate concentration in the serum of patients with schizophrenia. Procedia Chemistry..

[5] Ivanova SA, Smirnova LP, Shchigoreva YG, Semke AV, Bokhan NA (2015). Serum Glutathione in Patients with Schizophrenia in Dynamics of Antipsychotic Therapy. Bull Exp Biol Med..

[6] Sabherwal S, English JA, Föcking M, Cagney G, Cotter DR (2016). Blood biomarker discovery in drug-free schizophrenia: the contribution of proteomics and multiplex immunoassays. Expert Rev Proteomics..

[7] Saia-Cereda VM, Cassoli JS, Martins-de-Souza D, Nascimento JM (2017). Psychiatric disorders biochemical pathways unraveled by human brain proteomics. Eur Arch Psychiatry Clin Neurosci..

[8] Masopust J, Malý R, Andrýs C, Vališ M, Bažant J, Hosák L (2011). Markers of thrombogenesis are activated in unmedicated patients with acute psychosis: a matched case control study. BMC Psychiatry..

[9] Huang KC, Yang KC, Lin H, Tsao TT, Lee SA (2014). Transcriptome alterations of mitochondrial and coagulation function in schizophrenia by cortical sequencing analysis. BMC Genomics..

[10] Hoirisch-Clapauch S, Amaral OB, Mezzasalma MA, Panizzutti R, Nardi AE (2016). Dysfunction in the coagulation system and schizophrenia. Transl Psychiatry..

[11] Levin Y, Wang L, Schwarz E, Koethe D, Leweke FM, Bahn S (2010). Global proteomic profiling reveals altered proteomic signature in schizophrenia serum. Mol Psychiatry..

[12] Jaros JA, Martins-de-Souza D, Rahmoune H, Rothermundt M, Leweke FM, Guest PC, Bahn S (2012). Protein phosphorylation patterns in serum from schizophrenia patients and healthy controls. J Proteomics..

[13] Yang Y, Wan C, Li H, Zhu H, La Y, Xi Z, Chen Y, Jiang L, Feng G, He L (2006). Altered levels of acute phase proteins in the plasma of patients with schizophrenia. Anal Chem..

[14] Andreazza AC, Shao L, Wang JF, Young LT (2010). Mitochondrial complex I activity and oxidative damage to mitochondrial proteins in the prefrontal cortex of patients with bipolar disorder. Arch Gen Psychiatry..

[15] Lakhan SE (2012). Mass spectrometric analysis of prefrontal cortex proteins in schizophrenia and bipolar disorder. Springerplus..

[16] Du J, Machado-Vieira R, Khairova R (2011). Synaptic plasticity in the pathophysiology and treatment of bipolar disorder. Curr Top Behav Neurosci..

[17] Ohgi Y, Futamura T, Hashimoto K (2015). Glutamate Signaling in Synaptogenesis and NMDA Receptors as Potential Therapeutic Targetsfor Psychiatric Disorders. Curr Mol Med..

[18] Akarsu S, Torun D, Erdem M, Kozan S, Akar H, Uzun O (2015). Mitochondrial complex I and III mRNA levels in bipolar disorder. J Affect Disord..

[19] Levchuk L, Losenkov IS, Lebedeva EV, Schastnyy ED, Perchatkina OE, Bokhan NA (2017). Gene polymorphisms of enzymes biotransformation of xenobiotics in patients with depressive disorders. European Neuropsychopharmacology..

[20] Herberth M, Koethe D, Levin Y, Schwarz E, Krzyszton ND, Schoeffmann S, Ruh H (2011). Peripheral profiling analysis for bipolar disorder reveals markers associated with reduced cell survival. Proteomics..

[21] Schwarz E, Guest PC, Rahmoune H, Martins-de-Souza D, Niebuhr DW, Weber NS, Cowan DN, Yolken RH, Spain M, Barnes A, Bahn S (2012). Identification of a blood-based biological signature in subjects with psychiatric disorder prior to clinical manifestation. World J Biol Psychiatry..

[22] Song YR, Wu B, Yang YT, Chen J, Zhang LJ, Zhang ZW, Shi HY, Huang CL, Pan JX, Xie P (2015). Specific alterations in plasma proteins during depressed, manic, and euthymic states of bipolar disorder. Brazilian J Med Biol Res..

[23] Frye MA, Nassan M, Jenkins GD, Kung S, Veldic M, Palmer BA, Feeder SE, Tye SJ, Choi DS, Biernacka JM (2015). Feasibility of investigating differential proteomic expression in depression: implications for biomarker development in mood disorders. Transl Psychiatry..

[24] Alsaif M, Guest PC, Schwarz E, Reif A, Kittel-Schneider S, Spain M, Rahmoune H, Bahn S (2012). Analysis of serum and plasma identifies differences in molecular coverage, measurement variability, and candidate biomarker selection. Proteomics Clin Appl..

[25] Haenisch F, Cooper JD, Reif A, Kittel-Schneider S, Steiner J, Leweke FM, Rothermundt M, van Beveren NJM, Crespo-Facorro B, Niebuhr DW, Cowan DN, Weber NS, Yolken RH, Penninx BWJH, Bahn S (2016). Towards a blood-based diagnostic panel for bipolar disorder. Brain Behav Immun..

[26] Popova NM, Shakhurova NI, Schastnyy ED. Clinical features of affective disorders in the elderly. Eur Psychiatry. 2011;26(1):855.

[27] Preece RL, Han SYS, Bahn S (2018). Proteomic approaches to identify blood-based biomarkers for depression and bipolar disorders. Expert Rev Proteomics..

[28] Laemmli UK (1970). Cleavage of structural proteins during the assembly of the head of bacteriophage T4. Nature..

[29] Ishihama Y, Oda Y, Tabata T, Sato T, Nagasu T, Rappsilber J, Mann M (2005). Exponentially Modified Protein Abundance Index (emPAI) for Estimation of Absolute Protein Amount in Proteomics by the Number of Sequenced Peptides per Protein. Mol. Cell. Proteom..

[30] Ishihama Y, Schmidt T, Rappsilber J, Mann M, Hartl FU, Kerner MJ, Frishman D (2008). Protein abundance profiling of the *Escherichia coli* cytosol. BMC Genomics..

[31] Naryzhny SN, Zgoda VG, Maynskova MA, Novikova SE, Ronzhina NL, Vakhrushev IV, Khryapova EV, Lisitsa AV, Tikhonova OV, Ponomarenko EA, Archakov AI (2016). Combination of virtual and experimental 2DE together with ESI LC-MS/MS gives a clearer view about proteomes of human cells and plasma. Electrophoresis..

[32] Tusher VG, Tibshirani R, Chu G (2001). Significance analysis of microarrays applied to the ionizing radiation response. Proc. Natl. Acad. Sci. U.S.A..

[33] Dmitrieva EM, Smirnova LP, Loginova LV, Seregin AA, Dmitrieva EG, Ivanova SA (2014). The analysis of differences in electrophoretic the distribution of proteins in the blood serum of patients with schizophrenia and in healthy persons. Bulletin of Ural Medical Academic Science..

[34] Popova IG (2010). The role of endothelial dysfunction in the development of perinatal pathology in full-term newborns born to mothers with gestosis. PhD dissertation.

[35] Uzdensky A, Demyanenko S, Fedorenko G, Lapteva T, Fedorenko A (2017). Protein Profile and Morphological Alterations in Penumbra after Focal Photothrombotic Infarction in the Rat Cerebral Cortex. Mol Neurobiol..

[36] Dorofejeva O, Barr AJ (2017). Defining the molecular basis of interaction between R3 receptor-type protein tyrosine phosphatases and VE-cadherin. PLoS One..

[37] Rudimov EG, Buravkov SV, Andreeva EP, Buravkova LB (2015). Effect of proinflammatory activation on F-actin distribution in cultured human endothelial cells under conditions of experimental microgravity. Bull Exp Biol Med..

[38] Rudimov EG, Buravkova LB (2016). Gravisensitivity of endothelial cells: the role of cytoskeleton and adhesion molecules. Human Physiol..

[39] Sudakov NP, Klimenkov IV, Byvalt’sev VA, Nikiforov SB, Konstantinov YM (2017). Extracellular actin in health and disease. Biochemistry..

[40] Lei S, Czerwinska E, Czerwinski W, Walsh MP, MacDonald JF (2001). Regulation of NMDA Receptor Activity by F-Actin and Myosin Light Chain Kinase. J Neurosci..

[41] Yan Z, Kim E, Datta D, Lewis DA, Soderling SH (2016). Synaptic Actin Dysregulation, a Convergent Mechanism of Mental Disorders?. J Neurosci..

[42] Bhambhvani HP, Mueller TM, Simmons MS, Meador-Woodruff JH (2017). Actin polymerization is reduced in the anterior cingulate cortex of elderly patients with schizophrenia. Transl Psychiatry..

[43] Willemsen MH, Fernandez BA, Bacino CA, Gerkes E, de Brouwer AP, Pfundt R, Sikkema-Raddatz B, Scherer SW, Marshall CR, Potocki L, van Bokhoven H, Kleefstra T (2010). Identification of ANKRD11 and ZNF778 as candidate genes for autism and variable cognitive impairment in the novel 16q24.3 microdeletion syndrome. Eur J Hum Genet..

[44] Kambouris M, Maroun RC, Ben-Omran T, Al-Sarraj Y, Errafii K, Ali R, Boulos H, Curmi PA, El-Shanti H (2014). Mutations in zinc finger 407 [ZNF407] cause a unique autosomal recessive cognitive impairment syndrome. Orphanet J Rare Dis..

[45] Tao R, Cousijn H, Jaffe AE, Burnet PW, Edwards F, Eastwood SL, Shin JH, Lane TA, Walker MA, Maher BJ, Weinberger DR, Harrison PJ, Hyde TM, Kleinman JE (2014). Expression of ZNF804A in human brain and alterations in schizophrenia, bipolar disorder, and major depressive disorder: a novel transcript fetally regulated by the psychosis risk variant rs1344706. JAMA Psychiatry..

[46] Nielsen JA, Berndt JA, Hudson LD, Armstrong RC (2004). Myelin transcription factor 1 (Myt1) modulates the proliferation and differentiation of oligodendrocyte lineage cells. Mol Cell Neurosci..

[47] Zhu X, Mancini MA, Chang KH, Liu CY, Chen CF, Shan B, Jones D, Yang-Feng TL, Lee WH (1995). Characterization of a novel 350-kilodalton nuclear phosphoprotein that is specifically involved in mitotic-phase progression. Mol Cell Biol..

[48] Yang ZY, Guo J, Li N, Qian M, Wang SN, Zhu XL (2003). Mitosin/CENP-F is a conserved kinetochore protein subjected to cytoplasmic dynein-mediated poleward transport. Cell Res..

[49] Sato D, Lionel AC, Leblond CS (2012). SHANK1 Deletions in Males with Autism Spectrum Disorder. Am J Hum Genet..

[50] Andrade MA, Perez-Iratxeta C, Ponting CP (2001). Protein repeats: structures, functions, and evolution. J Struct Biol..

[51] Tang KS, Fersht AR, Itzhaki LS (2003). Sequential unfolding of ankyrin repeats in tumor suppressor p16. Structure (Camb)..

[52] Joutel A, Corpechot C, Ducros A, Vahedi K, Chabriat H, Mouton P, Alamowitch S, Domenga V, Cecillion M, Marechal E (1996). Notch3 mutations in CADASIL, a hereditary adult-onset condition causing stroke and dementia. Nature..

[53] Mosavi LK, Cammett TJ, Desrosiers DC, Peng ZY (2004). The ankyrin repeat as molecular architecture for protein recognition. Protein Sci..

[54] Ponce G, Hoenicka J, Jiménez-Arriero MA, Rodríguez-Jiménez R, Aragüés M, Martín-Suñé N, Huertas E, Palomo T (2008). DRD2 and ANKK1 genotype in alcohol-dependent patients with psychopathic traits: association and interaction study. Br J Psychiatry..

[55] Suraj Singh H, Ghosh PK, Saraswathy KN (2013). DRD2 and ANKK1 gene polymorphisms and alcohol dependence: a case-control study among a Mendelian population of East Asian ancestry. Alcohol Alcohol..

[56] Zhang A, Yeung PL, Li CW, Tsai SC, Dinh GK, Wu X, Li H, Chen JD (2004). Identification of a novel family of ankyrin repeats containing cofactors for p160 nuclear receptor coactivators. J Biol Chem..

[57] Sharma RP, Grayson DR, Gavin DP (2008). Histone deactylase 1 expression is increased in the prefrontal cortex of schizophrenia subjects: analysis of the National Brain Databank microarray collection. Schizophr Res..

[58] Benes FM, Lim B, Matzilevich D, Walsh JP, Subburaju S, Minns M (2007). Regulation of the GABA cell phenotype in hippocampus of schizophrenics and bipolars. Proc Natl Acad Sci U S A..

[59] Covington HE, Maze I, LaPlant QC, Vialou VF, Ohnishi YN, Berton O, Fass DM, Renthal W, Rush AJ, Wu EY, Ghose S, Krishnan V, Russo SJ, Tamminga C, Haggarty SJ, Nestler EJ (2009). Antidepressant actions of histone deacetylase inhibitors. J Neurosci..

[60] Kurita M, Holloway T, García-Bea A (2012). HDAC2 regulates atypical antipsychotic responses through the modulation of mGlu2 promoter activity. Nat Neurosci..

[61] Lo-Castro A, Brancati F, Digilio MC, Garaci FG, Bollero P, Alfieri P, Curatolo P (2013). Neurobehavioral phenotype observed in KBG syndrome caused by ANKRD11 mutations. Am J Med Genet B Neuropsychiatr Genet..

[62] Eker MC, Kitis O, Okur H, Eker OD, Ozan E, Isikli S, Akarsu N, Gonul AS (2011). Smaller hippocampus volume is associated with short variant of 5-HTTLPR polymorphism in medication-free major depressive disorder patients. Neuropsychobiology..

[63] Tsankova NM, Berton O, Renthal W, Kumar A, Neve RL, Nestler EJ (2006). Sustained hippocampal chromatin regulation in a mouse model of depression and antidepressant action. Nat Neurosci..

[64] Leussis MP, Madison JM, Petryshen TL (2012). Ankyrin 3: genetic association with bipolar disorder and relevance to disease pathophysiology. Biol Mood Anxiety Disord..

[65] Lopez AY, Wang X, Xu M, Maheshwari A, Curry D, Lam S, Adesina AM, Noebels JL, Sun QQ, Cooper EC (2017). Ankyrin-G isoform imbalance and interneuronopathy link epilepsy and bipolar disorder. Mol Psychiatry..

[66] Angst BD, Marcozzi C, Magee AI (2001). The cadherin superfamily: diversity in form and function. J Cell Sci..

[67] Yap AS, Kovacs EM (2003). Direct cadherin-activated cell signaling: a view from the plasma membrane. J Cell Biol..

[68] Dudek SM, Garcia JG (2001). Cytoskeletal regulation of pulmonary vascular permeability. J Appl Physiol (1985)..

[69] Liu Y, Chen XL, Wang L, Martins-Green M (2017). Insulin Antagonizes Thrombin-Induced Microvessel Leakage. J Vasc Res..

[70] Pang V, Bates DO, Leach L (2017). Regulation of human feto-placental endothelial barrier integrity by vascular endothelial growth factors: competitive interplay between VEGF-A165a, VEGF-A165b, PIGF and VE-cadherin. Clin Sci (Lond)..

[71] Gumbiner BM (2005). Regulation of cadherin-mediated adhesion in morphogenesis. Nat Rev Mol Cell Biol..

[72] Perez-Moreno M, Jamora C, Fuchs E (2003). Sticky business: orchestrating cellular signals at adherens junctions. Cell.

[73] Wang K, Zhang H, Ma D, Bucan M, Glessner JT (2009). Common genetic variants on 5p14.1 associate with autism spectrum disorders. Nature..

[74] Yagi T, Takeichi M (2000). Cadherin superfamily genes: functions, genomic organization, and neurologic diversity. Genes Dev..

[75] Singh SM, Castellani C, O'Reilly R (2010). Autism meets schizophrenia via cadherin pathway. Schizophr Res..

[76] Michaely P, Bennett V (1992). The ANK repeat: a ubiquitous motif involved in macromolecular recognition. Trends Cell Biol..

[77] Sedgwick SG, Smerdon SJ (1999). The ankyrin repeat: a diversity of interactions on a common structural framework. Trends Biochem Sci..

[78] Li J, Mahajan A, Tsai MD (2006). Ankyrin repeat: a unique motif mediating protein-protein interactions. Biochemistry..

[79] Marcotte EM, Pellegrini M, Eisenberg D, Yeates TO (1999). A census of protein repeats. J Mol Biol..

[80] Voronin DA, Kiseleva EV (2007). Functional Role of Proteins Containing Ankyrin Repeats. Tsitologiia..

[81] Levenson JM, O'Riordan KJ, Brown KD, Trinh MA, Molfese DL, Sweatt JD (2004). Regulation of histone acetylation during memory formation in the hippocampus. J Biol Chem..

[82] Gräff J, Joseph NF, Horn ME, Samiei A, Meng J, Seo J, Rei D, Bero AW, Phan TX, Wagner F, Holson E, Xu J, Sun J, Neve RL, Mach RH, Haggarty SJ, Tsai LH (2014). Epigenetic priming of memory updating during reconsolidation to attenuate remote fear memories. Cell..

[83] Guan JS, Haggarty SJ, Giacometti E, Dannenberg JH, Joseph N, Gao J, Nieland TJ, Zhou Y, Wang X, Mazitschek R, Bradner JE, DePinho RA, Jaenisch R, Tsai LH (2009). HDAC2 negatively regulates memory formation and synaptic plasticity. Nature..

[84] Gallagher D, Voronova A, Zander MA, Cancino GI, Bramall A, Krause MP, Abad C, Tekin M, Neilsen PM, Callen DF, Scherer SW, Keller GM, Kaplan DR, Walz K, Miller FD (2015). Ankrd11 is a chromatin regulator involved in autism that is essential for neural development. Dev Cell..

[85] McCarthy SE, Gillis J, Kramer M, Lihm J, Yoon S, Berstein Y, Mistry M, Pavlidis P, Solomon R, Ghiban E, Antoniou E, Kelleher E, O'Brien C, Donohoe G, Gill M, Morris DW, McCombie WR, Corvin A (2014). De novo mutations in schizophrenia implicate chromatin remodeling and support a genetic overlap with autism and intellectual disability. Mol Psychiatry..

[86] Mullin AP, Gokhale A, Moreno-De-Luca A, Sanyal S, Waddington JL, Faundez V (2013). Neurodevelopmental disorders: mechanisms and boundary definitions from genomes, interactomes and proteomes. Transl Psychiatry..

[87] Matsuyoshi N, Toda K, Horiguchi Y, Tanaka T, Nakagawa S, Takeichi M, Imamura S (1997). In vivo evidence of the critical role of cadherin-5 in murine vascular integrity. Proc Assoc Am Physicians..

[88] Pedrosa E, Stefanescu R, Margolis B, Petruolo O, Lo Y, Nolan K, Novak T, Stopkova P, Lachman HM (2008). Analysis of protocadherin alpha gene enhancer polymorphism in bipolar disorder and schizophrenia. Schizophr Res..

[89] Govorin NV, Vasilyeva AI (2011). Neuromarkers and indices of endothelial dysfunction in acute. Social and Clinical Psychiatry..

[90] Xu HB, Zhang RF, Luo D, Zhou Y, Wang Y, Fang L, Li WJ, Mu J, Zhang L, Zhang Y, Xie P (2012). Comparative proteomic analysis of plasma from major depressive patients: identification of proteins associated with lipid metabolism and immunoregulation. Int J Neuropsychopharmacol..

[91] Bot M, Chan MK, Jansen R, Lamers F, Vogelzangs N, Steiner J, Leweke FM, Rothermundt M, Cooper J, Bahn S, Penninx BW (2015). Serum proteomic profiling of major depressive disorder. Transl Psychiatry..

[92] Lee MY, Kim EY, Kim SH, Cho KC, Ha K, Kim KP, Ahn YM (2016). Discovery of serum protein biomarkers in drug-free patients with major depressive disorder. Prog Neuropsychopharmacol Biol Psychiatry..

[93] de Jesus JR, Galazzi RM, de Lima TB, Banzato CEM, de Almeida Lima E, Silva LF, de Rosalmeida Dantas C, Gozzo FC, Arruda MAZ (2017). Simplifying the human serum proteome for discriminating patients with bipolar disorder of other psychiatry conditions. Clin Biochem..

[94] Lee J, Joo EJ, Lim HJ, Park JM, Lee KY, Park A, Seok A, Lee H, Kang HG (2015). Proteomic analysis of serum from patients with major depressive disorder to compare their depressive and remission statuses. Psychiatry Investig..

[95] Chan MK, Cooper JD, Bot M, Steiner J, Penninx BWJH, Bahn S (2016). Identification of an Immune-Neuroendocrine Biomarker Panel for Detection of Depression: A Joint Effects Statistical Approach. Neuroendocrinology..

[96] Domenici E, Willé DR, Tozzi F, Prokopenko I, Miller S, McKeown A, Brittain C, Rujescu D, Giegling I, Turck CW, Holsboer F, Bullmore ET, Middleton L, Merlo-Pich E, Alexander RC, Muglia P (2010). Plasma protein biomarkers for depression and schizophrenia by multi analyte profiling of case-control collections. PLoS One..

[97] Demyanenko S, Uzdensky A (2017). Profiling of Signaling Proteins in Penumbra After Focal Photothrombotic Infarct in the Rat Brain Cortex. Mol Neurobiol..

[98] Kakurina GV, Kondakova IV, Choinzonov EL (2013). Proteomic patterns of biological fluids in patients with head and neck squamous cell carcinoma. Molecular Medicine..

[99] Kakurina GV, Kondakova IV, Cheremisina OV, Shishkin DA, Choinzonov EL (2015). Comparative study of blood serum proteins in patients with squamous cell head and neck cancer. Molecular Medicine..

