# Supplementary material for: The difference in serum proteomes in schizophrenia and bipolar disorder
Source: BMC Genomics. 2019 Jul 11;20(Suppl 7):535. doi: 10.1186/s12864-019-5848-1 (PMC6620192; doi:10.1186/s12864-019-5848-1)
Supplement: Supplementary file 1 — Approval by IRB-Ru. (PDF 3580 kb) [file 12864_2019_5848_MOESM1_ESM.pdf]

Федеральное государственное бюджетное научное учреждение
«Томский национальный исследовательский медицинский центр
Российской академии наук»
**НАУЧНО-ИССЛЕДОВАТЕЛЬСКИЙ ИНСТИТУТ
ПСИХИЧЕСКОГО ЗДОРОВЬЯ
(НИИ психического здоровья)**
Алеутская ул., д. 4, Томск, 634014, тел./ факс (3822) 72 43 79/ 72 44 25, e-mail: mental@tnimc.ru
ОКПО 01895186, ОГРН 1027000861568, ИНН/КПП 7019011979/701745005

24.09.2018 № 397/1

**ЛОКАЛЬНЫЙ ЭТИЧЕСКИЙ КОМИТЕТ
ПРИ ФЕДЕРАЛЬНОМ ГОСУДАРСТВЕННОМ БЮДЖЕТНОМ НАУЧНОМ УЧРЕЖДЕНИИ «ТОМСКИЙ НАЦИОНАЛЬНЫЙ ИССЛЕДОВАТЕЛЬСКИЙ МЕДИЦИНСКИЙ ЦЕНТР РОССИЙСКОЙ АКАДЕМИИ НАУК»
НАУЧНО-ИССЛЕДОВАТЕЛЬСКИЙ ИНСТИТУТ
ПСИХИЧЕСКОГО ЗДОРОВЬЯ
Томск, 634014, ул. Алеутская 4**

**Выписка из протокола заседания
Локального этического комитета при НИИ психического здоровья
№ 113 от 24 сентября 2018 года (Дело № 113/4.2018)**
Заседание состоялось по адресу: 634014 г. Томск, Алеутская 4,
НИИ психического здоровья
Дата поступления документов в ЛЭК при НИИ психического здоровья: 17.09.2018 г.
Председатель – д.м.н. Е.В. Гуткевич
Секретарь – д.б.н. В.Д. Прокопьева

Присутствуют:
к.м.н. Павлова О.А., д.м.н., профессор Куприянова И.Е., д.м.н., профессор Счастный Е.Д., юрисконсульт Кисель С.И., д.м.н., профессор Аксенов М.М., д.м.н., профессор Мандель А.И., Кириллова В.Ю., медицинская сестра, настоятель храма отец Алексий (Коновалов), сотрудник областной еженедельной газеты «Томские новости» Голикова Н.О., к.м.н. Мальцев В.С.

Повестка заседания:
об одобрении документов о подаче статьи “The difference in serum proteomes in schizophrenia and bipolar disorder” в журнал “Frontiers in Genetics, section Bioinformatics and Computational Biology”.

На рассмотрение представлены следующие документы:
1. Письмо-заявление в ЛЭК при НИИ психического здоровья от ответственного автора к.м.н. Л.П. Смирновой от 13.08.2018 г.;
2. Текст статьи на русском и английском языках;
3. Выписки из протоколов ЛЭКа при НИИ психического здоровья, г. Томск № 45 от 21 ноября 2011 года (Дело № 45/3.2011); № 90 от 23 мая 2016 года (Дело № 90/1.2016); № 93 от 24 октября 2016 года (Дело № 93/3.2016); № 109 от 23 апреля 2018 года (Дело № 109/4.2018); при ФГБНУ «НЦПЗ», г. Москва № 343 от 14 апреля 2017 года (Дело № 343.2017) с формами Информационных листков испытуемых и Краткими формами информированного согласия.
4. Заключения рецензентов – членов ЛЭКа д.м.н.. профессора М.М. Аксенова, д.м.н., профессора А.И. Мандель, юрисконсульта С.И. Кисель.

Итоговое заключение:
заседание проведено согласно положению о Локальном этическом комитете и стандартам Качественной Клинической Практики (GCP).

По представленным документам замечаний нет.

Принято решение одобрить представленные документы для подачи статьи “The difference in serum proteomes in schizophrenia and bipolar disorder” в журнал “Frontiers in Genetics, section Bioinformatics and Computational Biology”.

Ответственный автор: канд. мед. наук Л.П. Смирнова; клиническая база: «Томский национальный исследовательский медицинский центр Российской Академии наук», Научно-исследовательский институт психического здоровья (634014, Россия, г. Томск, ул. Алеутская 4).

Локальный этический комитет при НИИ психического здоровья организован и работает в соответствии с требованиями ICH GCP и законодательства Российской Федерации

Председатель ЛЭКа, д.м.н. Е.В. Гуткевич

Секретарь ЛЭКа, д.б.н. В.Д. Прокопьева

Подписи д.м.н. Е.В. Гуткевич и
д.б.н. В.Д. Прокопьевой
заверяю: ученый секретарь, к.м.н. Т.В. Казенных
