# Supplementary material for: The difference in serum proteomes in schizophrenia and bipolar disorder
Source: BMC Genomics. 2019 Jul 11;20(Suppl 7):535. doi: 10.1186/s12864-019-5848-1 (PMC6620192; doi:10.1186/s12864-019-5848-1)
Supplement: Supplementary file 2 — Approval by IRB.-Eng. (PDF 217 kb) [file 12864_2019_5848_MOESM2_ESM.pdf]

Federal state budgetary scientific institution
Tomsk National Research Medical Center of the Russian Academy of Sciences
Mental Health Research Institute
4 Aleutskaya Street, Tomsk, 634014, Russia
tel. (3822) 72 43 79/ 72 44 25, e-mail: mental@tnimc.ru
OKPO 01895186, OGRN 1027000861568, TIN/RRC 7019011979/701745005

ETHICAL COMMITTEE OF
TOMSK NATIONAL RESEARCH MEDICAL CENTER
OF THE RUSSIAN ACADEMY OF SCIENCES
MENTAL HEALTH RESEARCH INSTITUTE
4 Aleutskaya Street, Tomsk, 634014, Russia

Resolution of the Ethical Committee of Mental Health Research Institute
№ 113 from September 24, 2018 (№ 113/4.2018)
Address of the meeting: 4 Aleutskaya Street, Tomsk, 634014, Russia
Mental health research institute
Date of receipt of application at Ethical Committee of Mental health research institute: 17 September, 2018
Chairman - D.Sc. E.V. Gutkevich
Secretary - Doctor of Biological Sciences V.D. Prokopyeva

Present:
O.A. Pavlova, PhD; Professor I.E. Kuprijanova MD, DSc; Professor E.D. Schastnyi, MD, DSc; Attorney at Law S.I. Kissel; Professor M.M. Aksenov MD, DSc; Professor A.I. Mandel, MD, DSc; nurse V.Yu. Kirillova; V.S. Maltsev, PhD; Aleksij (Konovalov), local Religious Representative; N.O. Golikova, correspondent of the regional weekly newspaper “Tomsk News”.

Pavlova O.A. Ph.D.; Professor Kuprijanova I.E. M.D., D.Sc.;, Professor Schastnyi E.D. M.D., D.Sc., jurisconsult Kissel S.I.; M.D., D.Sc., Professor Aksenov M.M.; M.D., D.Sc., Professor Mandel A.I.; nurse Kirillova V.Yu.; Father Superior Aleksij (Konovalov); employee of the regional weekly newspaper “Tomskie novosti” Golikova N.O.; Ph.D. Maltsev V.S.

Meeting agenda:
Approval of submission of manuscript “The difference in serum proteomes in schizophrenia and bipolar disorder” to journal “Frontiers in Genetics, section Bioinformatics and Computational Biology”.

The following documents are submitted for consideration:
1. Letter of application to the Ethical Committee at the Mental Health Research Institute from the principal investigator L.P. Smirnova, Ph.D of 13 August, 2018;
2. Manuscript in English and Russian;
3. Resolutions of the Ethical Committee protocols of Mental health research institute , Tomsk № 45 of November 21, 2011 (№ 45/3.2011); № 90 of May 23, 2016 (№ 90/1.2016); № 93 of October 24, 2016 (№ 93/3.2016); № 109 of April 23, 2018 (№ 109/4.2018); FSBSI MFFRC, Moscow № 343 of April 14, 2017 (№ 343.2017) with the patient Informed Consent Form for the patients participating in the study;
4. Conclusion statement –Professor Aksenov M.M., M.D., D.Sc.,; Professor Mandel A.I., M.D., D.Sc.,; Kissel S.I., Attorney at Law

Summary decision:
The meeting was held according to the regulations of Ethical Committee and *Good Clinical Practice* standart.

Submitted documents were examined and considered sufficient.

It was decided to approve the manuscript submission “The difference in serum proteomes in schizophrenia and bipolar disorder” to journal “Frontiers in Genetics, section Bioinformatics and Computational Biology”.

The Principal Investigator: Smirnova, Ph.D. L.P.; Tomsk National Research Medical Center of the Russian academy of sciences, Mental Health Research Institute (4 Aleutskaya Street, Tomsk, 634014, Russia)

Ethical Committee of Mental Health Research Institute
was founded and acts in accordance with the requirements
of the ICH GCP and legislation Russian Federation

Chairman of the Ethical Committee D.Sc. E.V. Gutkevich

Secretary of the Ethical Committee Doctor of Biological Sciences V.D. Prokopyeva

Signatures of D.Sc. E.V. Gutkevich
and Doctor of Biological Sciences V.D. Prokopyeva
certified by academic secretary Ph.D. T.V. Kazennyh
