# Supplementary material for: The difference in serum proteomes in schizophrenia and bipolar disorder
Source: BMC Genomics. 2019 Jul 11;20(Suppl 7):535. doi: 10.1186/s12864-019-5848-1 (PMC6620192; doi:10.1186/s12864-019-5848-1)
Supplement: Supplementary file 3 — A statement regarding patient consent. (PDF 108 kb) [file 12864_2019_5848_MOESM3_ESM.pdf]

Informed consent statement

I hereby give my consent to participate in the research project «The difference in serum proteomes in schizophrenia and bipolar disorder».

Responsible executive: Liudmila Smirnova, MD, PhD.

Address: 634014 Tomsk, 4 Aleutskaya Street. Telephone: +7 (3822) 72-43-79

I am aware of the following statements:

* The examination is still experimental

* The objectives of this study

* Possible risks

I have received the above information in writing and got answers to all my questions.

I give my consent to the investigators to review my medical record, provided ensure the professional confidentiality.

I am aware that I have the right to terminate my participation in the study anytime and receive appropriate testing and treatment.

I agree to the fact that the information about me is entered into legally protected database. I have the right to access these data and, if necessary, correct it with the doctor.

Full name of patient: ______________________

Date ________________________

Signature ________________________

Full name of investigator: Liudmila Smirnova

Date ________________________

Signature ________________________

Address: 634014 Tomsk, 4 Aleutskaya Street. Telephone: +7 (3822) 72-43-79
